# PromA Plasmids Are Instrumental in the Dissemination of Linuron Catabolic Genes Between Different Genera

**DOI:** 10.3389/fmicb.2020.00149

**Published:** 2020-02-18

**Authors:** Johannes Werner, Eman Nour, Boyke Bunk, Cathrin Spröer, Kornelia Smalla, Dirk Springael, Başak Öztürk

**Affiliations:** ^1^Department of Biological Oceanography, Leibniz Institute for Baltic Sea Research, Rostock, Germany; ^2^Institute for Epidemiology and Pathogen Diagnostics, Julius Kühn-Institut, Federal Research Centre for Cultivated Plants (JKI), Braunschweig, Germany; ^3^Faculty of Organic Agriculture, Heliopolis University for Sustainable Development, Cairo, Egypt; ^4^Bioinformatics Department, Leibniz Institute DSMZ, German Collection of Microorganisms and Cell Cultures, Braunschweig, Germany; ^5^Central Services, Leibniz Institute DSMZ, German Collection of Microorganisms and Cell Cultures, Braunschweig, Germany; ^6^Division of Soil and Water Management, KU Leuven, Leuven, Belgium; ^7^Junior Research Group Microbial Biotechnology, Leibniz Institute DSMZ, German Collection of Microorganisms and Cell Cultures, Braunschweig, Germany

**Keywords:** broad-host-range plasmids, horizontal gene transfer, biodegradation, transposases, plasmid ecology

## Abstract

PromA plasmids are broad host range (BHR) plasmids, which are often cryptic and hence have an uncertain ecological role. We present three novel PromA γ plasmids which carry genes associated with degradation of the phenylurea herbicide linuron, two of which originated from unrelated *Hydrogenophaga* hosts isolated from different environments (pPBL-H3-2 and pBPS33-2), and one (pEN1) which was exogenously captured from an on-farm biopurification system (BPS). *Hydrogenophaga* sp. plasmid pBPS33-2 carries all three necessary gene clusters (*hylA, dca, ccd*) determining the three main steps for conversion of linuron to Krebs cycle intermediates, while pEN1 only determines the initial linuron hydrolysis step. *Hydrogenophaga* sp. plasmid pPBL-H3-2 exists as two variants, both containing *ccd* but with the *hylA* and *dca* gene modules interchanged between each other at exactly the same location. Linuron catabolic gene clusters that determine the same step were identical on all plasmids, encompassed in differently arranged constellations and characterized by the presence of multiple IS*1071* elements. In all plasmids except pEN1, the insertion spot of the catabolic genes in the PromA γ plasmids was the same. Highly similar PromA plasmids carrying the linuron degrading gene cargo at the same insertion spot were previously identified in linuron degrading *Variovorax* sp. Interestingly, in both *Hydrogenophaga* populations not every PromA plasmid copy carries catabolic genes. The results indicate that PromA plasmids are important vehicles of linuron catabolic gene dissemination, rather than being cryptic and only important for the mobilization of other plasmids.

## Introduction

Plasmids are circular or linear extrachromosomal elements that can self-replicate, and are important agents in the dissemination of genes among microbial species (Garcillan-Barcia et al., 2011). Broad host range (BHR) plasmids can independently transfer and maintain themselves in different taxa (Jain and Srivastava, 2013) and carry accordingly genes for replication, maintenance and control, and conjugation (Szpirer et al., 1999). In addition, BHR plasmids may carry so-called “accessory” genes, for instance for antibiotic and heavy metal resistance, or biodegradation of xenobiotic compounds (Schlüter et al., 2007; Sen et al., 2011). On the other hand, exogenous plasmid capture enabled the isolation of several plasmids with few or no apparent accessory genes that belong to known BHR plasmid groups such as IncP-1, IncN and IncU from environmental microbial communities (Brown et al., 2013). These so far cryptic plasmids have no apparent benefit to the host and are still propagated in absence of selective pressure (Fox et al., 2008). A recently discovered group of BHR plasmids are the PromA plasmids, most of which were isolated by exogenous plasmid capture (Schneiker et al., 2001; Tauch et al., 2002; Van der Auwera et al., 2009; Li et al., 2014; Thomas et al., 2017; Yanagiya et al., 2018), hence originated from unknown hosts. Exogenous plasmid isolation allows capturing conjugative as well as mobilizable plasmids from environmental microorganisms by means of biparental and triparental matings without the need to cultivate the host (Smalla et al., 2015). A few PromA plasmids also originated from proteobacterial isolates (Ito and Iizuka, 1971; Mela et al., 2008; Van der Auwera et al., 2009). With the exception of pSFA231 (Li et al., 2014), pMOL98 (Van der Auwera et al., 2009), and pSB102 (Schneiker et al., 2001) which carry heavy metal resistance genes, all 12 completely sequenced PromA plasmids identified to date are cryptic plasmids with no clear indication of their ecological role or possible benefit for the host organism. It was hypothesized that the main role of these plasmids is to mobilize other plasmids (Zhang et al., 2015).

Recently, we have described two PromA plasmids, pPBL-H6-2 and pPBS-H4-2 from two *Variovorax* strains with the metabolic capability to degrade the phenylurea herbicide linuron (Öztürk et al., 2019). *Variovorax* is a genus that is isolated at high frequency from enrichments that use linuron as sole source of carbon energy. In the linuron-degrading *Variovorax* sp. strains isolated to date, the initial step of linuron degradation to 3,4-dichloroaniline (DCA) is performed by the linuron amidases encoded by the *hylA* or *libA* genes, followed by the *dcaQA1A2BR* catabolic cluster coding for the enzymes that convert DCA to 4,5-dichlorocatechol. The catechol intermediate is further degraded to Krebs cycle intermediates by the enzymes encoded by the *ccdCFDER* gene cluster (Bers et al., 2011, 2013). The two *Variovorax* PromA plasmids belong to the PromA γ subgroup together with the plasmids pSN1104-11 and pSN1104-34 (Yanagiya et al., 2018) that were exogenously isolated from the microbial granules of an anaerobic waste water treatment plant. pPBS-H4-2 is the first PromA plasmid that carries catabolic genes, i.e., it carries a stretch of DNA containing the *hylA* gene and the *ccd* cluster and several IS*1071* elements, of which two border the cargo at both ends. In contrast, pPBL-H6-2 only contains one copy of IS*1071* as cargo. The catabolic genes of pPBS-H4-2 were identical to those of *Variovorax* sp. WDL1. In WDL1, the catabolic genes are located on a megaplasmid (pWDL1-1) rather than on PromA plasmids. Two mutually-exclusive versions of this plasmid occur in the WDL1 population, one carrying the *hylA* gene and the *ccd* cluster that are encompassed by separate IS*1071* insertion elements, and the other carrying the *dca* cluster instead of the *hylA* gene, again flanked by IS*1071* insertion elements (Albers et al., 2018; Öztürk et al., 2019).

In this study, we report the characterization of three new linuron catabolic PromA γ plasmids. In contrast to pPBL-H6-2 and pPBS-H4-2, these plasmids did not originate from *Variovorax*. Two of the new plasmids were isolated from two different linuron degrading *Hydrogenophaga* strains, PBL-H3 and BPS33. *Hydrogenophaga* sp. strain PBL-H3 was isolated from a potato field near Halen, Belgium (Breugelmans et al., 2007) and *Hydrogenophaga* sp. strain BPS33 from the matrix of an on farm biopurification system (BPS) operated by Inagro, near Roeselare, Belgium, in this study. The genus *Hydrogenophaga* belongs to the family of *Comamonadaceae*, and was formerly classified as *Pseudomonas* (Willems et al., 1989). Although some members of this genus are marked by their ability to oxidize hydrogen (Willems et al., 1989), *Hydrogenophaga* is not a genus that is frequently associated with xenobiotic degradation. Exceptions comprise *Hydrogenophaga intermedia* S1 and PMC, which mineralize 4-aminobenzenesulfonate in two-species consortia (Gan et al., 2011) and the pyrene-degrading *Hydrogenophaga* sp. PYR1 (Yan et al., 2017). The third new PromA plasmid is plasmid pEN1, which was obtained by biparental exogenous isolation from a BPS operating on a farm near Kortrijk, Belgium (Dealtry et al., 2016), selecting for mercury resistant transconjugants of the recipient strain. Here, we compare the full sequences of three new plasmids with each other and with other previously reported PromA plasmids including those discovered in the linuron degrading *Variovorax* in order to deduce their evolution and the role that PromA plasmids play in the dissemination of linuron degradation genes in different genera and environments.

## Materials and Methods

### Chemicals

Linuron ([3-(3,4-dichlorophenyl)-1-methoxy-1-methyl urea] PESTANAL^®^, analytical standard) was purchased from Sigma Aldrich. [phenyl-U-14C] Linuron (16.93 mCi mmol^–1^, radio- chemical purity > 95%) was purchased from Izotop.

### Isolation of *Hydrogenophaga* sp. BPS33

BPS33 was isolated from the matrix of a BPS located at research institute Inagro in Rumbeke-Beitem, Belgium (50°54′07.9″N 3°07′28.2″E). The BPS had received linuron and other pesticides for 2 years. The sample was collected from the upper 10 cm of the top container, and stored at 4°C until further use. The isolation procedure followed the protocol previously described by Breugelmans et al. (2007). Briefly, 1 g of the matrix material was inoculated into 50 mineral medium MMO (Dejonghe et al., 2003) containing 20 mg/L linuron. Degradation of linuron was monitored using HPLC as described before (Horemans et al., 2014). After linuron was degraded, dilutions of the enrichment culture were plated on R2A medium (Breugelmans et al., 2007) containing 20 mg/L linuron. Resulting colonies were inoculated into 2.5 mL 96-well plates containing 500 μL of MMO with 20 mg/L linuron, and colonies that degraded linuron were identified via 16S rRNA gene sequencing with primers 27F and 1492R (primers listed in Supplementary Table S2). Both BPS33 and PBL-H3 used in this study were routinely cultivated in R2B medium supplemented with 20 mg/L linuron. Prior to genome sequencing, the linuron mineralization capacity of both cultures was determined as described before (Breugelmans et al., 2007). Each mineralization test contained 10^8^ colony forming units of BPS33 or PBL-H3 in 40 mL MMO medium and a total radioactivity of 0.009 mCi mL^–1^.

### Isolation of pEN1 by Exogenous Capture

Plasmid pEN1 was exogenously captured from BPS material (collected from Kortrijk, Belgium) spiked with linuron in a microcosm experiment as described by Dealtry et al. (2016). In brief, *Pseudomonas putida* KT2442:*gfp* was used as a recipient strain for plasmids conferring mercury chloride resistance (20 μg mL^–1^). Biparental mating was performed with a bacterial suspension extracted from the BPS matrix 25 days after linuron spiking.

### Genome Sequencing

DNA was isolated using Qiagen Genomic-tip 100/G (Qiagen, Hilden, Germany) according to the instructions of the manufacturer. SMRTbell^TM^ template library was prepared according to the instructions from PacificBiosciences, Menlo Park, CA, United States, following the Procedure and Checklist – Greater Than 10 kbp Template Preparation. Briefly, for preparation of 15 kbp libraries 8 μg genomic DNA and 1.4 μg plasmid DNA was sheared using g-tubes^TM^ from Covaris, Woburn, MA, United States, according to the manufacturer’s instructions. DNA was end-repaired and ligated overnight to hairpin adapters applying components from the DNA/Polymerase Binding Kit P6 from Pacific Biosciences, Menlo Park, CA, United States. Reactions were carried out according to the manufacturer’s instructions. For the bacterial DNAs BluePippin^TM^ Size-Selection to greater than 4 kbp was performed according to the manufacturer’s instructions (Sage Science, Beverly, MA, United States). Conditions for annealing of sequencing primers and binding of polymerase to purified SMRTbell^TM^ template were assessed with the Calculator in RS Remote, PacificBiosciences, Menlo Park, CA, United States. One SMRT cell was sequenced per strain/plasmid on the PacBio RSII (PacificBiosciences, Menlo Park, CA, United States) taking one 240 min movies.

For the bacterial DNA libraries for sequencing on Illumina platform were prepared applying Nextera XT DNA Library Preparation Kit (Illumina, San Diego, CA, United States) with small modifications (Baym et al., 2015). Samples were sequenced on NextSeq^TM^ 500.

Bacterial long read genome assemblies were performed applying the RS_HGAP_Assembly.3 protocol included in SMRT Portal version 2.3.0 applying target genome sizes of 10 Mbp. For BPS33, the genome assembly directly revealed the chromosomal and both plasmid sequences. In case of PBL-H3, the assembly revealed the chromosomal sequence misassembled together with the 107 kbp plasmid. Thus, this plasmid sequence was separated from the chromosome and processed independently. Nevertheless, the 319 kbp plasmid was detected as separate contig. Further artificial contigs with low coverage and/or included in other replicons were removed from the assembly. All remaining contigs were circularized; particularly assembly redundancies at the ends of the contigs were removed. Replicons were adjusted to *dnaA* (chromosome) or *repA-parA* (all plasmids) as the first gene. Error-correction was performed by a mapping of the Illumina short reads onto finished genomes using bwa v. 0.6.2 in paired-end (sampe) mode using default setting (Li and Durbin, 2009) with subsequent variant and consensus calling using VarScan v. 2.3.6 (parameters: mpileup2cns –min-coverage 10 –min-reads2 6 –min-avg-qual 20 –min-var-freq 0.8 –min-freq-for-hom 0.75 –*p*-value 0.01 –strand-filter 1 –variants 1 –output-vcf 1) (Koboldt et al., 2012). A consensus concordance of QV60 could be reached. Automated genome annotation was carried out using Prokka v. 1.8 (Seemann, 2014). The *hylA*-containing plasmid pPBL-H3-2 was assembled using a target genome size of 200 kbp. Interestingly, only 25% of the plasmid population was shown to carry the transposon-based insertion based on plasmid coverage analysis.

### Assembly of pPBL-H3-2 Variants B2 and B4

The pPBL-H3-2 variants were assembled using the pBPS33-2 as a scaffold, as these two plasmids carried identical catabolic genes, and the pBPS33-2 carried a correctly assembled complete *dca* cluster. The PBL-H3 Illumina paired-end reads were mapped to pBPS33-2 using BWA-MEM v. 0.7.17.1 with standard settings (Li and Durbin, 2009) implemented in the Galaxy platform (Afgan et al., 2016). A consensus of the *dca* cluster genes together with the flanking regions was generated using samtools v. 2.1.4 mpileup with standard settings (Li et al., 2009). This consensus was aligned to pPBL-H3-2 (B2). The flanking regions matched pPBL-H3-2 (B2) perfectly, with *hylA* and the associated genes being located between these flanking sequences instead of the *dca* cluster. The consensus sequence with the *dca* cluster, acquired from the mpileup was inserted in the place of the *hylA* cluster in pPBL-H3-2 (B2) to obtain pPBL-H3-2 (B4). The new plasmid was annotated as described before. The assembly procedure is illustrated in Supplementary Figure S1.

### Comparative Genomics Analysis

Both phylogenetic trees and dDDH values were computed on the Type (Strain) Genome Server (TYGS) (Meier-Kolthoff and Goker, 2019). In brief, the TYGS analysis was subdivided into the following steps: The 16S rRNA gene sequences were extracted from the genomes using RNAmmer (Lagesen et al., 2007). All pairwise comparisons among the set of genomes were conducted using GBDP and intergenomic distances inferred under the algorithm “trimming” and distance formula d_5_ (Meier-Kolthoff et al., 2013). Hundred distance replicates were calculated. Digital DDH values and confidence intervals were calculated using the recommended settings of the GGDC 2.1 (Meier-Kolthoff et al., 2013). The resulting intergenomic distances were used to infer a balanced minimum evolution tree with branch support via FASTME 2.1.4 including SPR postprocessing (Lefort et al., 2015). Branch support was inferred from 100 pseudo-bootstrap replicates each. The trees were rooted at the midpoint and visualized with iTOL (Letunic and Bork, 2019). For constructing the phylogenetic trees, six type strains and six additional *Hydrogenophaga* genomes were used to determine the phylogenetic position of the two linuron-degrading *Hydrogenophaga* strains within the genus. A dDDH species cutoff of 70% was applied as described before (Liu et al., 2015). The list of genomes included in the study is given in Supplementary Table S1.

The alignment of the PromA plasmids was performed with AliTV v. 1.0.6 (Ankenbrand et al., 2017). Codon usage frequencies were calculated with Comparem v 0.0.23 (Parks, 2018), the PCA with R v. 3.5.2 (R Core Team, 2019) and FactoMineR v. 1.41 (Lê et al., 2008).

The plasmid sequences were categorized under known plasmid groups based on the amino acid and nucleotide identity of their backbone genes to known plasmids, using BLAST against the NCBI nr database (Altschul et al., 1990). To elucidate the phylogeny of the PromA plasmids, Proteinortho v. 6.0.11 (Lechner et al., 2011) was used to extract the plasmid core proteins from all PromA plasmids sequenced to date. The core amino acid sequences [RepA, ParA, Ssb, TrbB (VirB11), TraG (VirD4), TraC (VirB4), TraK (VirB9), TraB (VirB10), TraL (VirB3), TrbJ (VirB8), TraE (VirB5), TraA (VirB1), RepB, KorA, TraS (VirD2), TrbC (VirB2), ArdC] were concatenated and aligned with MAFFT v. 7.453 (Katoh and Standley, 2013) and the maximum likelihood (ML) tree was calculated with RaxML v. 8.2.12 (Stamatakis, 2014) using the under the PROTCATAUTO model and 1000 bootstrap replicates. The tree was rooted at midpoint and visualized with iTOL (Letunic and Bork, 2019). The genomic locations of the IS*1071* elements and catabolic genes were determined and the genes were visualized using Geneious v. 11.0.4.

### Quantification of 16S rRNA Gene, Plasmids, and Catabolic Genes by Real-Time Quantitative PCR (qPCR)

The PCR and qPCR primer sequences used in this study are listed in Supplementary Table S2. For the qPCR analysis, the strains BPS33 and PBL-H3 were grown to OD_600_ as described above. DNA extraction was made from 2 mL of culture as previously described (Larsen et al., 2007). Each reaction contained 10 ng of template DNA. qPCR reactions were performed with the ABsolute QPCR Mix (Thermo Fisher) on a Roche LightCycler 480 II. The qPCRs for 16S rRNA gene (Lopez-Gutierrez et al., 2004), *hylA* (Horemans et al., 2016), and *dcaQ* (Albers et al., 2018) quantification were performed as previously described. The qPCR targeting *korB* was performed, as previously described, except that the Taqman probe was omitted (Jechalke et al., 2013). Each PCR reaction to generate the templates for the qPCR standard curves contained 10 ng template DNA (BPS33 gDNA), 1× Dream Taq Green buffer (Thermo Fisher), 0.2 M of each dNTP, 0.1 μM of each primer and 1.25 U of Dream Taq DNA polymerase (Thermo Fisher) in a final volume of 50 μl. The amplification was performed as follows: Initial denaturation of 95°C for 3 min, 40 cycles of denaturation at 95°C for 30 s, annealing at 60°C for 30 s, extension at 72°C for 1 min, followed by a final extension at 72°C for 15 min. All conventional PCR reactions were performed with an Applied Biosystems Veriti 96-well thermal cycler. The products were purified with the DNA Clean & Concentrator 25 kit (Zymo) and quantified with the Qubit BR DNA assay (Thermo Fisher Scientific).

## Results

### Phylogenetic Analysis of the PromA Plasmids

*Hydrogenophaga* plasmids pPBL-H3-2, pBPS33-2, and exogenously captured pEN1, were completely sequenced. The general features of plasmids pEN1, pPBL-H3-2, and pBPS33-2 are given in Table 1.

**TABLE 1:** Statistics of the genomes and plasmids sequenced in this study.

	Contig size	#CDS	%GC	# rRNA operons	#tRNA	Classification
pEN1	59 kbp	68	63.4			PromA plasmid
*chr* BPS33	6.33 Mbp	5,830	65.7	2	53	Chromosome
pBPS33-1	340 kbp	323	61.2			Unclassified plasmid
pBPS33-2	107 kbp	109	63.1			PromA plasmid
*chr* PBL-H3	4.39 Mbp	4.39	65.6	2	44	Chromosome
pPBL-H3-1	320 kbp	320	61.1			Unclassified plasmid
pPBL-H3-2	107 kbp	107	62.8		1	PromA plasmid

**FIGURE 1:**
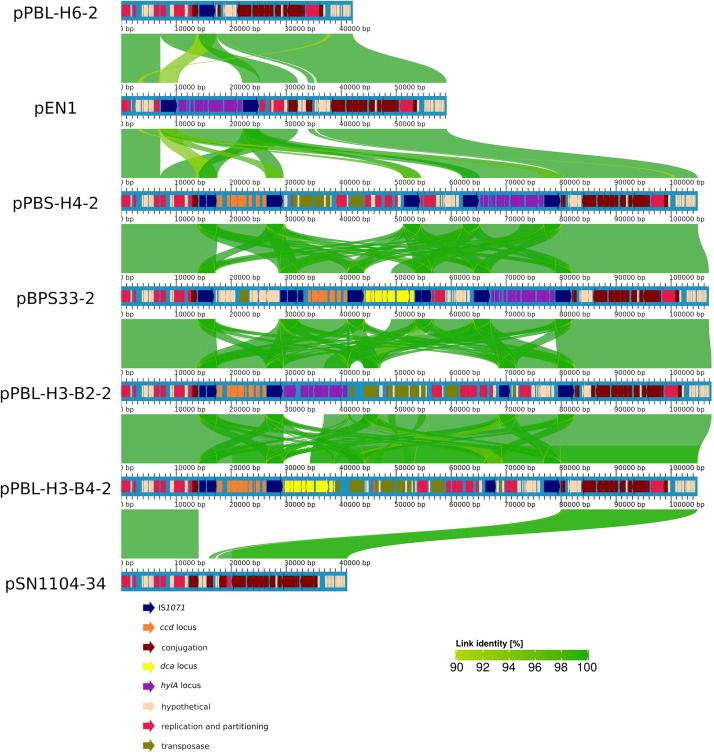
Alignment of PromA γ plasmids. Alignment identities are shown for an identity of 90–100%. Each plasmid is aligned to the one above.

**FIGURE 2:**
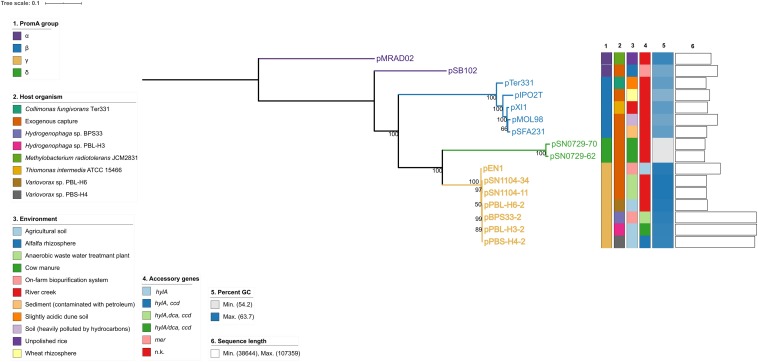
Core protein-based phylogeny of PromA plasmids. The branches are scaled in terms of the expected number of substitutions per site. The numbers above the branches are support values when larger than 60% from ML. n.k., not known.

A whole-sequence alignment revealed that the plasmids pEN1, pBPS33-2, and pPBL-H3-2 have a high sequence identity and synteny to the previously described plasmids pPBS-H4-2 and pPBL-H6-2 from linuron-degrading *Variovorax* sp. (Öztürk et al., 2019; Figure 1). The core protein-based phylogenetic analysis showed that these plasmids all belong to the PromA γ group, together with the plasmids pSN1104-11 and pSN1104-34 (Yanagiya et al., 2018; Figure 2).

**FIGURE 3:**
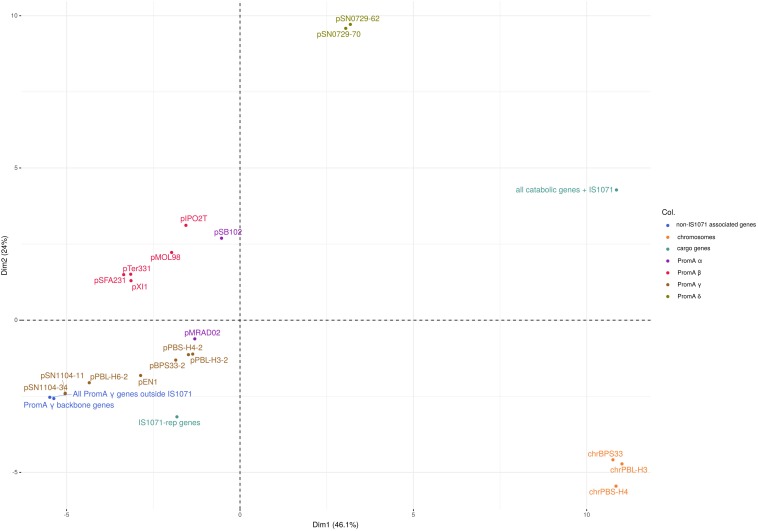
PCA of the codon usage frequencies of the PromA plasmids. PromA γ plasmids were included as whole plasmids, as well as only backbone genes (PromA γ backbone genes), all genes excluding cargo genes (All PromA γ genes outside IS*1071*), IS*1071*-associated replication/partitioning genes (IS*1071*-*rep* genes), IS*1071*-associated catabolic genes including the IS*1071* TnpA (all catabolic genes + IS*1071*) as well as the three PromA γ host chromosomes.

The *repA* sequences, as well as the conjugal-transfer-related gene sequences of the PromA γ plasmids were highly conserved, with 99% nucleotide sequence identity. Codon usage-based clustering showed that the catabolic PromA γ plasmids clustered together and separately from the non-catabolic ones (Figure 3 and Supplementary Table S3). The average codon usage of the plasmid backbone genes of pPBL-H3-2 B2 and B4, pBPS33-2, and pPBS-H4-2 was significantly different to that of their respective catabolic clusters. When the IS*1071* elements were removed from the catabolic plasmid sequences, these clustered with other PromA γ plasmids. It was also observed that the codon usage of the hosts (BPS33, PBS-H4, and PBL-H3) were different from these of both the cargo genes and plasmid backbone genes. The codon usage profiles of different PromA groups did not always follow the phylogeny, as the closely related PromA γ and δ group plasmids had a significantly different codon usage.

### Catabolic Potential of the PromA Plasmids

**FIGURE 4:**
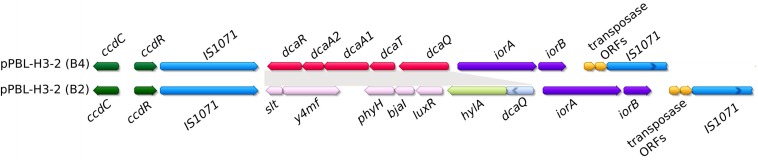
Comparison of catabolic clusters between plasmids pPBL-H3-2 (B4) and (B2). Shaded regions represent the ORFs that are different between the two plasmids. Broken arrows indicate truncated ORFs.

The newly sequenced PromA plasmids pBPS33-2, pPBL-H3-2, and pEN1 all carry genes related to linuron degradation. After the assembly of the PBL-H3 genome, it was observed that the genome did not contain the *dca* cluster, which was not expected due to the capacity of this strain to mineralize linuron. A BLAST search against the unassembled PacBio reads indeed revealed the presence of the same *dca* cluster as the one present on pBPS33-2 in the PBL-H3 genome. The new assembly, which makes use of the similarity between the two plasmids as described in the Materials and Methods section, revealed that PBL-H3 had two different versions of the pPBL-H3-2 within the population, named as pPBL-H3-2 (B2) and pPBL-H3-2 (B4). Both pPBL-H3-2 (B2) and pPBL-H3-2 (B4) are identical except that the pPBL-H3-2 (B4) locus carrying *hylA* gene and associated open reading frames (ORFs), is replaced by the *dca* gene cluster and associated ORFs (Figure 4). Plasmids pPBL-H3-2 (B2) and (B4) both carry the *ccdCFDER* genes that are 99% identical to those previously identified in *Variovorax* sp. WDL1 and PBS-H4. Both loci have 99% nucleotide identity and complete synteny to their WDL1/PBS-H4 counterparts.

Plasmid pBPS33-2 carries all the genes necessary for linuron degradation. The *hylA*, *dcaQA1A2BR*, and *ccdCFDER* genes have 99% nucleotide identity and complete synteny to those previously identified in *Variovorax* sp. WDL1 and PBS-H4. The plasmid pEN1 on the other hand only contains *hylA* and associated ORFs. The *hylA* gene on all plasmids truncates a *dcaQ* gene, with the junction being identical to the *hylA*-*dcaQ* junction in pPBS-H4-2 (Öztürk et al., 2019). In contrast, pEN1 carries a *hylA*-*dcaQ* junction identical to that on pWDL1-1, in *Variovorax* sp. WDL1 (Albers et al., 2018; Öztürk et al., 2019). As previously reported for pWDL1-1 and pPBS-H4-2, the catabolic clusters on the newly sequenced PromA plasmids are flanked by IS*1071* insertion elements, forming putative composite transposons. In pBPS33-2, the *ccd* and *dca* clusters are adjacent to each other, with one IS*1071* in between. The IS*1071* element associated with *hylA* is separated from the *dca* and *ccd* and flanked by two additional IS*1071* elements. In all cases, IS*1071* are complete and contain inverted repeats (IRs) at both ends of the IS*1071* transposases.

In addition to the catabolic clusters related to linuron degradation, pBPS33-2 carries an extra IS*1071* element flanking genes that encode for four proteins putatively involved in the *meta*-pathway of phenol degradation and three putative multidrug efflux pump proteins, with an intermittent single copy of an IS*91* insertion sequence, encompassing 19 kbp in total (pBPS33-2_13860__–__33156_).

### PromA Plasmid Associated IS*1071* Insertion Elements and Their Genomic Organization

**FIGURE 5:**
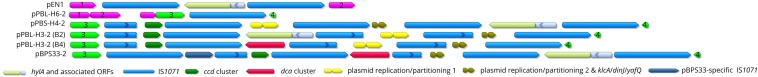
The synteny of the IS*1071* insertion sites on various PromA γ plasmids. The bases between the IS*1071* elements were deleted for simplicity and the IS*1071*-associated cargo was represented by single ORFs, functions of which are indicated in the legend. Broken arrows represent truncated IS*1071 tnpA* ORFs. The IS*1071*-flanking plasmid backbone genes are numbered 1–4: (1) *repB* (2) *mobA* (3) *trbM* (4) *traS*. Light green arrows represent conjugation-related genes, and purple arrows represent replication/partitioning genes.

The catabolic PromA plasmids have a high number of IS*1071* elements, some of which are associated with the catabolic clusters. IS*1071* elements are absent in the non-catabolic PromA plasmids, with the exception of pPBL-H6-2 (Öztürk et al., 2019). The IS*1071* element sequences are largely similar to the classical structure (Sota et al., 2006); except that some elements seem to code for truncated transposases due to a premature stop codon (Figure 5). Plasmid pBPS33-2 has six IS*1071* elements, the highest number among all PromA plasmids described so far, followed by five in pPBS-H4-2, four in pPBL-H3-2, two in pEN1 and one in pPBL-H6-2.

The first IS*1071* insertion sites relative to *repA* among the plasmids pBPS33-2, pPBL-H3-2, and pPBS-H4-2 are the same, being adjacent to the plasmid mobility genes *mobC* and *traS* and the 3′ end of the last IS*1071* element being flanked by the backbone *trbM* gene (Figure 5 and Supplementary Figure S2). pPBL-H6-2 contains a single IS*1071* element without cargo genes. For pBPS33-2, pPBL-H3-2 (B2/B4), and pPBS-H4-2, the insertion site has multiple IS*1071* insertions, with multiple catabolic clusters and other accessory genes inserted consecutively separated by an IS*1071* element including IRs. Interestingly, some genes that related to DNA replication and plasmid maintenance functions made also part of the IS*1071* associated cargo. These are genes encoding the plasmid replication initiation proteins RepA and RepB (two ORFs, named *repB1* and *repB2*) (100% amino acid identity to WP_068682750.1, WP_068682748.1, and WP_068682748.1, respectively), segregation protein ParB (100% amino acid identity to WP_011114060.1) and tyrosine recombinase XerC (100% amino acid identity to WP_068682758.1) on the first IS*1071*-flanked region (present in pPBL-H3-2 (B2/B4) and pBPS-H4-2) and toxin/antitoxin system proteins KlcA/DinJ/YafQ (100% amino acid identity to WP_011114069.1, WP_011114070.1, and WP_011114071.1, respectively), segregation protein ParA (100% amino acid identity to CDS81791.1) and plasmid partitioning proteins KorB (100% amino acid identity to WP_011114060.1) and KfrA (truncated copy) on the second IS*1071*-flanked region [present in pBPS33-2, pPBL-H3-2 (B2/B4), and pBPS-H4-2] (Supplementary Figure S2). The non-catabolic PromA γ plasmids lack the IS*1071*-associated plasmid replication-related genes as well as the toxin/antitoxin system genes altogether. On the other hand, all PromA γ plasmids have copies of *repA, parA* and *parB* genes, on their backbone which are not flanked by IS*1071* elements and are unrelated to those flanked by the IS*1071* elements on the catabolic plasmids. In addition to these accessory genes, six putative transposases and thirteen hypothetical proteins were found in the IS*1071*-flanked region in pPBS-H4-2 and pPBL-H3-2 (B2/B4), which are conserved among those plasmids. Six ORFs encoding hypothetical proteins are present in pBPS33-2. These are absent in the other PromA γ plasmids. The first IS*1071*-associated putative replication/segregation gene cluster as well as the transposase homologs align at 99% nucleotide identity with the entire length of the unclassified 24 kbp plasmid pWDL1-4 (Supplementary Figure S3) identified in the linuron-degrading *Variovorax* sp. WDL1 (Öztürk et al., 2019). Plasmid pWDL1-4 carries one copy of IS*1071* with a small insertion at position 1092, resulting in a truncated TnpA. The second IS*1071*-associated locus aligned in its full length to various IncP-1 group plasmids such as pAKD33 (JN106174) (Sen et al., 2011) and pTT11 (MH392242.1). On pTT11, these genes were also preceded by an IS*1071* transposase. In case of the plasmids pPBL-H3-2 (B2/B4), pPBS33-2 and pPBS-H4-2, however, the *kfrA* gene was interrupted at position 396 by an IS*1071* element, which was present in its full length in the IncP-1 plasmids.

The insertion site of the IS*1071* composite transposon carrying the *hylA* locus in pEN1 is an exception among the PromA plasmids. This transposon lies between the backbone genes *repB* and *mobA*. This site is located before the first insertion site of the other catabolic plasmids relative to *repA*, and exists in other PromA γ plasmids, but with four nucleotide differences. The left IR of the first IS*1071* element on pEN1 has twelve nucleotide differences to the previously described IS*1071* left IR (Sota et al., 2006). The left IR of the second IS*1071* on pEN1, just like all the other left IRs on the other catabolic PromA plasmids, is identical to what was previously described (Sota et al., 2007).

In pPBL-H3-2 (B2/B4), the IS*1071* element flanking the right side of the *hylA/dca* catabolic cluster encodes a truncated variant of the IS*1071* transposase, which is not the case in pBPS33-2 and pPBS-H4-2, where both IS*1071* transposases are intact (Figure 5). On PBL-H3-2 (B2/B4) and pBPS33-2 the *ccd* cluster is adjacent to the *hylA*/*dca* clusters, with one IS*1071* element in between. The left flanking IS*1071* transposase of the *ccd* cluster appears truncated in all PromA plasmids at identical nucleotide positions. In all cases, truncations were caused by an immature stop codon as a result of a point mutation. This truncated transposase was not present in the pWDL1-1 of *Variovorax* sp. WDL1.

### PromA Plasmid and Catabolic Gene Copy Numbers in *Hydrogenophaga*

As there were two variants of the pPBL-H3-2 present in the PBL-H3 population, the question arose whether each version is represented in equal numbers, and how this compares to the BPS33 population where only one plasmid version could be assembled. The copy numbers of the genes encoding KorB (single copy plasmid backbone gene), HylA, DcaQ, as well as 16S rRNA were determined to elucidate both the number of PromA plasmid copies per cell and the proportions of PromA plasmids that carry the catabolic genes. Cultures of PBL-H3 and BPS33 contained approximately 10 copies of the PromA plasmids per cell. The *hylA* and *dcaQ* gene copy numbers in both strains were similar, at about one copy per 100 cells, i.e., about one copy per 1000 PromA plasmid copies.

### General Genome Characteristics and Phylogeny

The general genome characteristics of the two *Hydrogenophaga* strains are summarized in Table 1. Prior to sequencing, the ability of both strains to mineralize linuron was confirmed as described in the Materials and Methods section. Both genomes comprise one chromosome and two plasmids.

To the date of this publication, 22 *Hydrogenophaga* genomes have been deposited to GenBank, five of which are complete genomes. The complete list of genomes included in the phylogenetic analysis is given in Supplementary Table S1. The full genomes have a size of 4.39–6.32 Mbp, with GC contents ranging from 61 to 70%. BPS33 has the largest genome of them with 6.32 Mbp. This is an exceptional size, *Hydrogenophaga* genome next to it being 5.23 Mbp. Apart from the linuron-degrading strains sequenced in this study, only *Hydrogenophaga pseudoflava* DSM 1084 (pDSM1084, NZ_CP037868.1, 45.2 kbp) contains plasmids.

**FIGURE 6:**
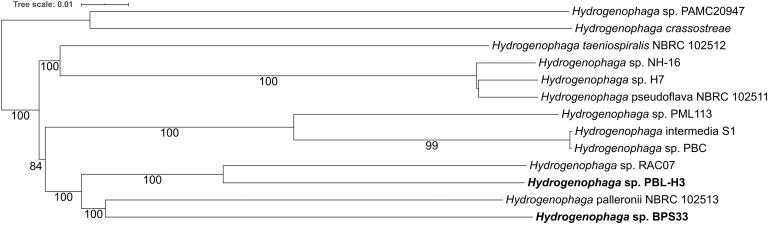
Phylogeny of *Hydrogenophaga* sp. strains based on fully sequenced genomes based on whole genome sequences. The linuron-degrading *Hydrogenophaga* sp. PBL-H3 and BPS33 sequenced in this study are marked in bold. The branch lengths are scaled in terms of GBDP distance formula *d5*. The numbers above branches are GBDP pseudo-bootstrap support values > 60% from 100 replications.

Phylogenetic analysis revealed a distant relation of PBL-H3 and BPS33 to each other (Figure 6 and Supplementary Figure S4). The computed DNA-DNA hybridization of 25.7% confirms that these two isolates are different species. The isolates do not belong to any type species.

Both *Hydrogenophaga* strains carry megaplasmids, pBPS33-1 and pPBL-H3-1, in addition to the PromA plasmids, which could not be assigned to any known plasmid class. The plasmids do not share any similarity and have no similarity to megaplasmids previously identified in *Variovorax* (Öztürk et al., 2019) or any other plasmid from *Hydrogenophaga*.

## Discussion

In this study, we have investigated the genetic basis of linuron degradation by the two *Hydrogenophaga* strains PBL-H3 and BPS33, and the role of PromA plasmids in the dissemination of catabolic genes in different environments. This study builds up on the previous finding that PromA plasmids can carry catabolic genes as opposed to being solely cryptic plasmids (Öztürk et al., 2019). We have described possible hot spots for transposon insertion, and the origin of non-catabolic IS*1071*-associated genes located on these plasmids. The genomes of two linuron-degrading *Hydrogenophaga* strains were completely sequenced and chromosomes and plasmids were circularized. These strains were sampled from two different environments, and although they belong to the same genus, they are distantly related to each other. The linuron-degrading *Variovorax* strains were very closely related to each other despite belonging to different species (Öztürk et al., 2019). Within the genus *Hydrogenophaga*, however, the ability to acquire xenobiotic degradation genes seems to be independent of the host phylogeny.

The PromA plasmids pPBL-H3-2 and pBPS33-2 are the sole carriers of linuron-related catabolic clusters in both strains analyzed in this study. This was also the case for *Variovorax* sp. PBS-H4, although this strain lacks the *dca* cluster genes that are required for complete linuron mineralization (Öztürk et al., 2019). The catabolic PromA plasmids have a nearly identical backbone to the previously described PromA γ plasmids isolated in Japan (Yanagiya et al., 2018). It has been reported before that BHR plasmids isolated from different geographic locations can be highly conserved (Heuer et al., 2004; Chen et al., 2015; Li et al., 2016), however, some degree of divergence in the backbone structures of PromA plasmid groups were reported before (Li et al., 2014). In this case, the nearly identical backbone genes indicate that these plasmids had a recent common ancestor, probably without any accessory genes. Interestingly, the PromA plasmids of *Hydrogenophaga* sp. PBL-H3 (B2) and *Variovorax* sp. PBS-H4, which were isolated from the same soil sample, have a high synteny of their cargo genes and are 99% identical over their entire length including the backbone genes, indicating that these plasmids can possibly be transferred between these two genera in the same environment. The codon usage distribution of the catabolic genes is significantly different to the codon usage of the backbone genes, and the codon usage of neither the plasmids nor the catabolic genes seem to be adapted to their host. This emphasizes the mobile character of these elements.

IS*1071* elements were associated with all catabolic clusters related to linuron degradation on all plasmids. Remarkably, all catabolic clusters were nearly identical to what has previously been described for *Variovorax* (Öztürk et al., 2019), both in terms of gene identity and synteny. This demonstrates that even among different genera and environments, the linuron degradation pathways rely on a limited genetic repertoire, and that the role of IS*1071* elements to transfer these genes is not limited to a certain genus or environment. The cargo associated by IS*1071* elements on these plasmids, however, were not limited to catabolic genes. Putative plasmid backbone genes involved in plasmid replication and maintenance as well as hypothetical proteins were also associated with IS*1071* elements, which were absent in the PromA plasmids without IS*1071* elements. The nearest relatives of the plasmid backbone genes associated with IS*1071* elements originated from different organisms and plasmids, among which are IncP-1 plasmids (*parAB*) but also non-linuron-degrading *Variovorax* chromosomes (*repAB*). Interestingly, two composite transposons flanked by IS*1071* elements on plasmids pPBL-H3-2 (B2/B4), pPBS-H4-2 and one on pBPS33-2 show high synteny and similarity to the backbones of previously sequenced plasmids. Cointegration of replicons was reported to be the first step in IS*1071* transposition, which is then resolved by the host’s recombination system (Nakatsu et al., 1991; Ng and Wyndham, 1993). It is therefore possible that the catabolic PromA plasmids evolved by cointegrating IS*1071* elements and associated clusters from other catabolic plasmids, and that the IS*1071*-associated replication/partitioning genes are the remnants of such a recombination event.

The identical cargo insertion sites on the PromA γ plasmids indicate hot spots for transposon insertion. Hot spots were previously reported for IncP-1 plasmids (Thorsted et al., 1996; Sota et al., 2007; Dunon et al., 2018). The insertion of transposons at specific sites might contribute to plasmid stability (Sota et al., 2007). The hot spot on our PromA γ plasmids is located between the genes encoding the relaxase TraS and conjugal transfer protein TrbM, which is different from the *parA* locus that was previously shown to be the insertion site of pSFA231 (Li et al., 2014), pMOL98 (Van der Auwera et al., 2009), and pSB102 (Schneiker et al., 2001), as well using PCR-based methods in environmental DNA (Dias et al., 2018).

Different to other catabolic PromA plasmids, pPBL-H3-2 existed as two variants, one carrying the *hylA* gene and associated ORFs, and the other the *dca* cluster. Isogenic subpopulations carrying either *hylA* or *dca* loci were reported for *Variovorax* sp. WDL1 before (Albers et al., 2018). It was proposed that the existence of two subpopulations may be an adaptation to linuron degradation in a consortium, where linuron is degraded to DCA by the *hylA*-carrying *Variovorax*, while the DCA degradation is performed by the other consortium members (Albers et al., 2018). Indeed, WDL1 degrades linuron less efficiently on its own than when it is a part of a consortium (Dejonghe et al., 2003). Like WDL1, PBL-H3 also tended to accumulate DCA when growing on linuron on its own, but performed much better in a consortium with other DCA degraders (Breugelmans et al., 2007). Thus, this *Hydrogenophaga* strain might be adapted to degrading linuron in a consortium in a similar way to WDL1. The major difference between WDL1 and PBL-H3 is that, while with WDL1 the majority of the population has either the *hylA* gene or the *dca* cluster (Albers et al., 2018), subpopulations containing either gene are much underrepresented in the PBL-H3 population.

The *hylA* gene and *dca* cluster carrying PromA plasmids are underrepresented by almost 100-fold compared to the total number of PromA plasmids in both *Hydrogenophaga* species. The mobile elements associated with these catabolic clusters seem to be unstable, and are lost from most of the PromA plasmid copies in both populations. The loss of catabolic clusters from plasmids, both in the presence and absence of other C sources than the xenobiotic compound, was shown in various studies (Takahashi et al., 2009; Changey et al., 2011; Horemans et al., 2017). In case of plasmid pADP1 from *Pseudomonas huttiensis*, it was observed that the loss of a 47 kbp upstream atrazine catabolic region via an IS*Pps1*-mediated homologous recombination event led to an overall fitness increase in the host when growing on the atrazine downstream degradation product cyanuric acid. This was proposed to be due to the loss of a burden constituted by this region, either by lessening the cost of plasmid replication or by reducing the energetic cost of basal gene expression (Changey et al., 2011). It is also possible in the case of PromA plasmids that the loss of an inessential catabolic region during growth on complex media increases host fitness, causing the non-degrading members of the community to overgrow the degrading ones.

The results show that even among different genera, the genes for complete linuron mineralization are highly conserved, being acquired through horizontal gene transfer which is mediated by BHR plasmids. PromA γ plasmids, in addition to the previously known IncP-1 plasmids, are carriers of IS*1071* elements and associated catabolic pathways, being present in different contaminated ecosystems. In contrast to the linuron-degrading *Variovorax* species, where the degradation genes are also found on megaplasmids as well as BHR plasmids (Öztürk et al., 2019), the *Hydrogenophaga* catabolic genes are only found on BHR plasmids, pointing toward a more recent acquisition of these gene clusters. The results in addition demonstrate that the PromA γ plasmids can possibly be transferred between the two different genera, *Hydrogenophaga* and *Variovorax* in the same environment.

## Data Availability Statement

The datasets generated for this study can be found in the BPS33 genome is available under the GenBank accession numbers CP044549-CP044551, PBL-H3 (B2) under CP044975-CP044977, PBL-H3 (B4) under CP044972-CP044974, and pEN1 plasmid sequence under MN536506.

## Author Contributions

BÖ designed the study and performed the experiments on the *Hydrogenophaga* strains. BÖ and JW performed the sequence analysis of the *Hydrogenophaga* genomes and comparative analysis of PromA plasmids. BB and CS performed the sequencing and assembly of all genomes and plasmids. EN and KS isolated pEN1 and performed the sequence analysis. BÖ, JW, and DS wrote the main body of the manuscript. All authors contributed to the writing and critical reading of this publication.

## Conflict of Interest

The authors declare that the research was conducted in the absence of any commercial or financial relationships that could be construed as a potential conflict of interest.
