# Supplementary material for: PromA Plasmids Are Instrumental in the Dissemination of Linuron Catabolic Genes Between Different Genera
Source: Front Microbiol. 2020 Feb 18;11:149. doi: 10.3389/fmicb.2020.00149 (PMC7039861; doi:10.3389/fmicb.2020.00149)
Supplement: Supplementary file 1 [file Data_Sheet_1.docx]

## Supplementary information

Supplementary table S1: list of *Hydrogenophaga* genomes used for the taxonomic analysis

| Organism | Accession nr | Genome size (Mbp) |
| --- | --- | --- |
| Hydrogenophaga pseudoflava NBRC 102511 | GCF_001592285.1 | 4.5 |
| Hydrogenophaga palleronii NBRC 102513 | GCF_001571225.1 | 4.84 |
| Hydrogenophaga crassostreae LPB0072T | GCF_001761385.1 | 4.94 |
| Hydrogenophaga pseudoflava NBRC 102511 | GCF_001592285.1 | 4.5 |
| Hydrogenophaga sp. NH-16 | GCF_004006415.1 | 4.83 |
| Hydrogenophaga sp. RAC07 | GCF_001713375.1 | 4.67 |
| Hydrogenophaga intermedia S1 | GCF_000723405.1 | 5.28 |
| Hydrogenophaga sp. PML113 | GCF_001777155.1 | 4.7 |
| Hydrogenophaga taeniospiralis NBRC 102512 | GCF_001592305.1 | 5.25 |
| Hydrogenophaga sp. PBC | GCF_000263795.2 | 5.23 |
| Hydrogenophaga sp. PAMC20947 | GCF_004795855.1 | 4.85 |
| Hydrogenophaga sp. H7 | GCF_002025745.1 | 4.49 |
| Hydrogenophaga sp. PBL-H3 | This study | 4.39 |
| Hydrogenophaga sp. BPS33 | This study | 6.32 |

Supplementary table S2: Primers used in this study

| **Plasmid name** | **Sequence (5‘-3‘)** | **Reference** | **Use/target** |
| --- | --- | --- | --- |
| **HylA-RT-F** | GCATGGGTCTGTTGCTGATAC | (Horemans *et al.*, 2016) | qPCR/*hylA* gene |
| **HylA-RT-R** | CTGCGTGGAACTTCACTGTTAG | (Horemans *et al.*, 2016) | qPCR/*hylA* gene |
| **W-31** | GTACCTGCTGGAGAACCACCT | (Albers *et al.*, 2018) | qPCR/*dcaQ* gene |
| **W-32** | GGAACAGCACCATCGTGTCC | (Albers *et al.*, 2018) | qPCR/*dcaQ* gene |
| **Eub341F** | CCTACGGGAGGCAGCAG | (Muyzer *et al.*, 1993) | qPCR/16S rRNA gene |
| **Eub534R** | ATTACCGCGGCTGCTGG | (Muyzer *et al.*, 1993) | qPCR/16S rRNA gene |
| **korB-RT-F** | TCATCGACAACGACTACAACG | (Jechalke *et al.*, 2013) | qPCR/*korB* gene |
| **korB-RT-R** | TTCTTCTTGCCCTTCGCCAG | (Jechalke *et al.*, 2013) | qPCR/*korB* gene |
| **linhydrWDL1_comp_Fw** | TCATTTCGCCGGCTCGCCAA | (Bers *et al.*, 2013) | PCR/*hylA* gene |
| **linhydrWDL1_comp_Rv** | ATGCCGATGCATAGGGCCATAT | (Bers *et al.*, 2013) | PCR/*hylA* gene |
| **W1-33** | TGCTGGCCGACCTTTACATGA | (Albers *et al.*, 2018) | PCR/*dcaQ* gene |
| **W1-34** | CTACAAAGCCTTGGATGGCGG | (Albers *et al.*, 2018) | PCR/*dcaQ* gene |
| **27F** | AGAGTTTGATCCTGGCTCAG | (Polz and Cavanaugh, 1998) | PCR/16S rRNA gene |
| **1492R** | GGTTACCTTGTTACGACTT | (Polz and Cavanaugh, 1998) | PCR/16S rRNA gene |
| **korB-F** | ATGAGCGCCAAGACCAACGC | This study | PCR/*korB* gene |
| **korB-R** | TCAGCCCTCCAGCAGCG | This study | PCR/*korB* gene |


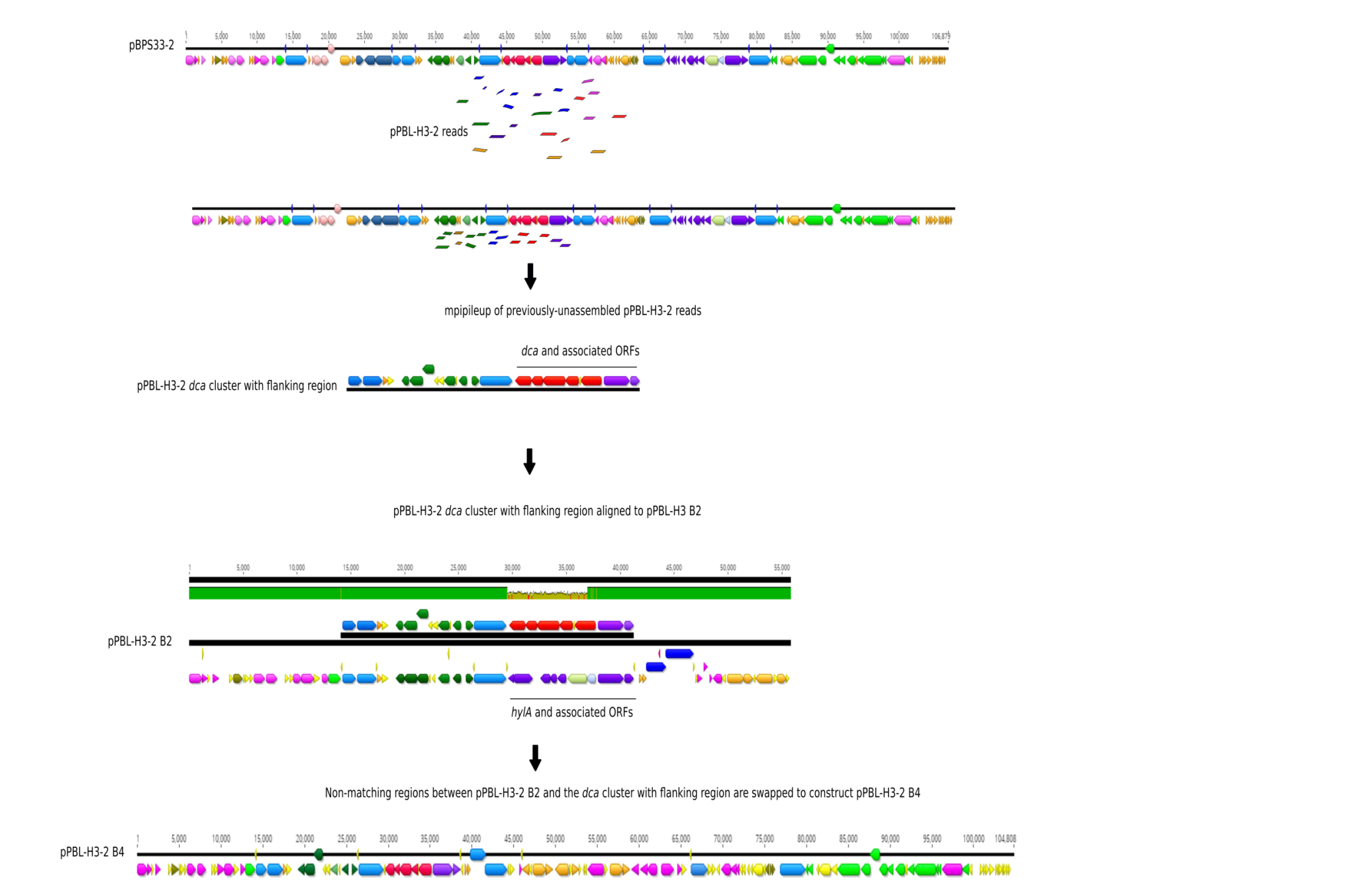


Supplementary figure S1: Assembly of pPBL-H3-2 B4 using pBPS33 as a reference. The image was generated with Geneious v. 11.0.4 and edited with Inkscape.


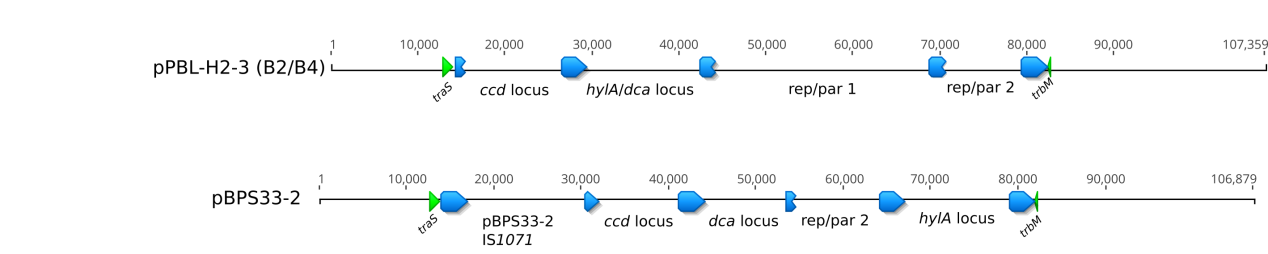


A


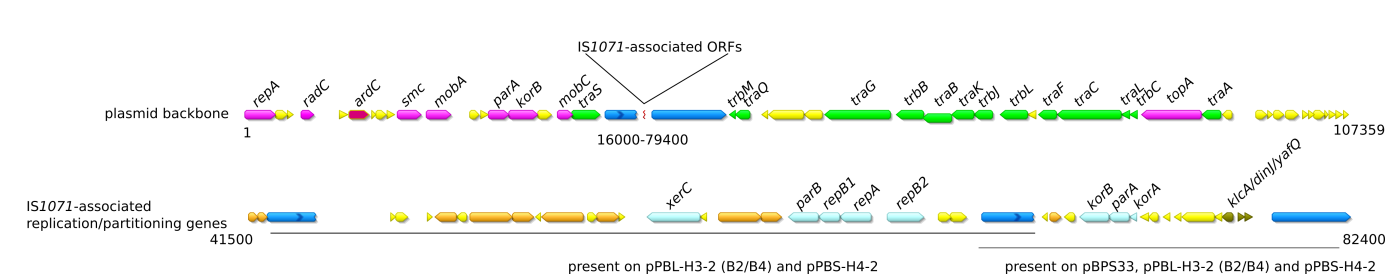


B

Supplementary figure S2: A) Insertion sites of the various IS*1071* elements on the plasmids pPBL-H2-2 (B2/B4) and pBPS33-2 B) Organization of the plasmid backbone genes as well as IS*1071* associated plasmid replication/partitioning genes on catabolic PromA plasmids pPBL-H3-2 (B2/B4), pPBS-H4-2 and pBPS33-2. The region which were conserved in all four and the region which was only present in three plasmids (pPBL-H3-2 (B2/B4) and pPBS-H4-2) were marked on the figure. Dark blue arrows represent IS*1071* elements, purple: replication/partitioning genes, yellow: hypothetical proteins; orange: miscellaneous transposases; green: conjugation-related genes. The image was generated with Geneious v. 11.0.4 and edited with Inkscape.


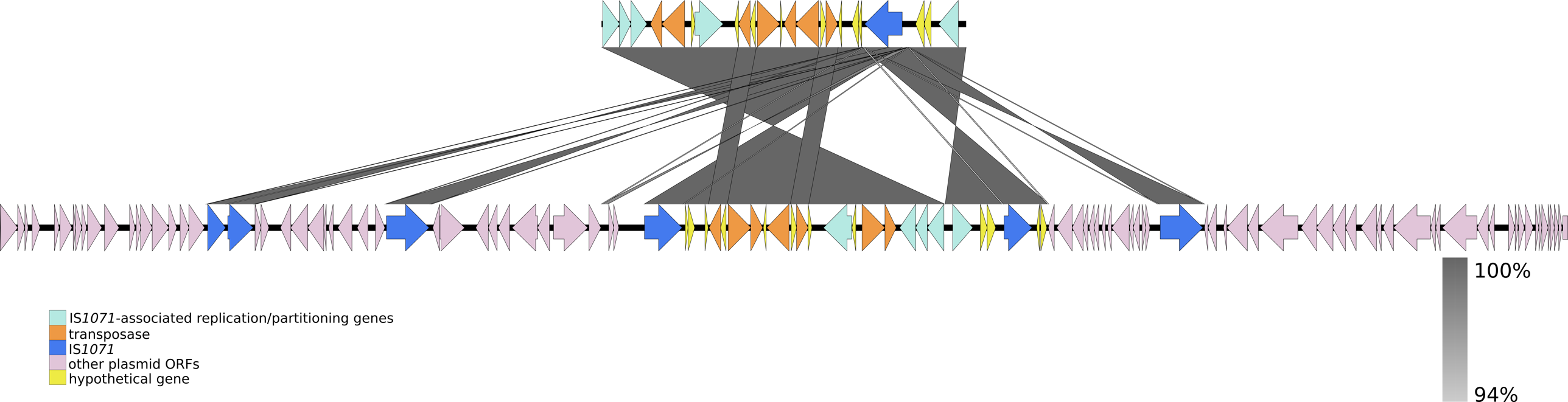


Supplementary figure S3: Alignment of pWDL1-4 to pPBL-H3-2 (B2). As the plasmids pPBL-H3 (B2/B4) and pPBS-H4-2 had identical IS*1071*-associated replication/partitioning genes, only one representative alignment was shown. The figure was generated with Easyfig v. 2.3.3 (Sullivan *et al.*, 2011).


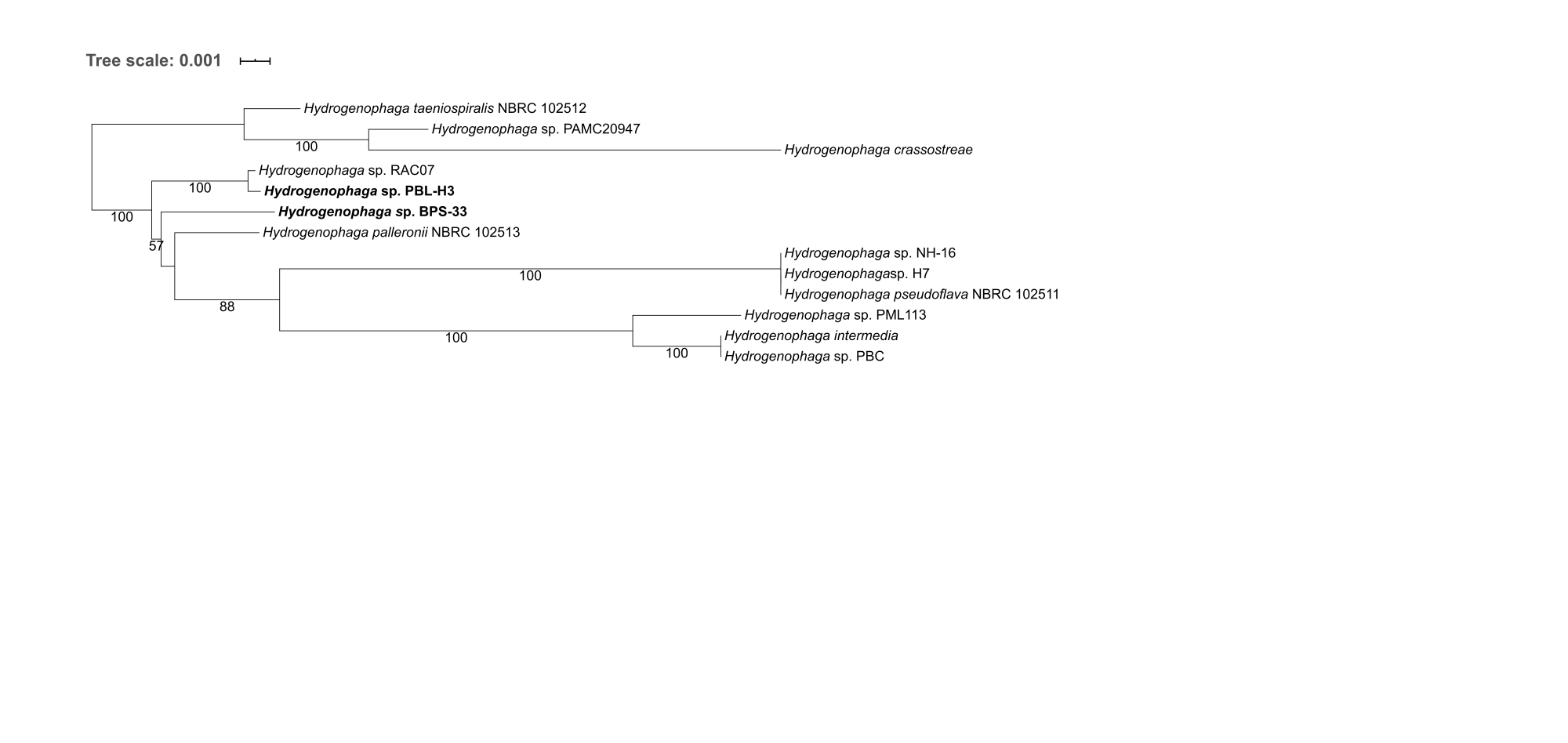


Supplementary figure S4: 16S rRNA gene sequence-based phylogeny of *Hydrogenophaga* species. The linuron-degrading *Hydrogenophaga* sp. PBL-H3 and BPS33 sequenced in this study are marked in bold. The branch lengths are scaled in terms of GBDP distance formula d5. The numbers above branches are GBDP pseudo-bootstrap support values > 60% from 100 replications*.*

Albers, P., Lood, C., Özturk, B., Horemans, B., Lavigne, R., van Noort, V., et al. (2018) Catabolic task division between two near-isogenic subpopulations co-existing in a herbicide-degrading bacterial consortium: consequences for the interspecies consortium metabolic model. *Environ Microbiol* **20**: 85–96.

Bers, K., Batisson, I., Proost, P., Wattiez, R., De Mot, R., and Springael, D. (2013) Hyla, an alternative hydrolase for initiation of catabolism of the phenylurea herbicide linuron in variovorax sp. strains. *Appl Environ Microbiol* **79**: 5258–5263.

Horemans, B., Bers, K., Ruiz Romero, E., Pose Juan, E., Dunon, V., De Mot, R., and Springael, D. (2016) Functional Redundancy of Linuron Degradation in Microbial Communities in Agricultural Soil and Biopurification Systems. *Appl Environ Microbiol* **82**: 2843–2853.

Jechalke, S., Dealtry, S., Smalla, K., and Heuer, H. (2013) Quantification of IncP-1 plasmid prevalence in environmental samples. *Appl Environ Microbiol* **79**: 1410–1413.

Muyzer, G., de Waal, E.C., and Uitterlinden, A.G. (1993) Profiling of complex microbial populations by denaturing gradient gel electrophoresis analysis of polymerase chain reaction-amplified genes coding for 16S rRNA. *Appl Environ Microbiol* **59**: 695–700.

Polz, M.F. and Cavanaugh, C.M. (1998) Bias in template-to-product ratios in multitemplate PCR. *Appl Environ Microbiol* **64**: 3724–3730.
